# Characterization of Hospitalized Patients With Dengue Fever in Can Tho City: A Cross‐Sectional Analysis, 2018‐2019

**DOI:** 10.1155/cjid/6410209

**Published:** 2026-02-25

**Authors:** Thuy Thi Thu Nguyen, Phuong Vu Mai Hoang, Hang Le Khanh Nguyen, Hau Thi Bich Vu, Phuc Van Tran, Truc Minh Huynh, Hung Minh Ha, Thanh Huy Ong, K. Morita, F. Hasebe, Mai Thi Quynh Le

**Affiliations:** ^1^ Virology Department, National Institute of Hygiene and Epidemiology, Hanoi, Vietnam, nihe.org.vn; ^2^ Infectious Diseases Department, Can Tho City General Hospital, Can Tho City, Vietnam; ^3^ Department of Infectious Disease Control and International Health Quarantine, Can Tho Centre for Disease Control and Prevention, Can Tho City, Vietnam; ^4^ Infectious Diseases Department, Can Tho Pediatric Hospital, Can Tho City, Vietnam; ^5^ Virology Department, Institute of Tropical Medicine, Nagasaki University, Nagasaki City, Japan, nagasaki-u.ac.jp

**Keywords:** dengue fever in Can Tho: warning signs, hospitalized patients, secondary infections

## Abstract

Dengue poses a significant public health issue in tropical and subtropical countries, especially in urban areas. Can Tho, the fourth‐largest city in Vietnam’s Mekong Delta, is highly vulnerable to climate change. This study characterizes dengue fever (DF) and identifies factors linked to severe disease in hospitalized patients from 2018 to 2019. We analyzed the clinical manifestations of 123 patients, along with blood count tests, RT‐PCR, the NS1 antigen rapid test, and ELISA for IgM and IgG antibodies, upon admission. The results revealed that 88.6% of dengue patients were under 15 years, primarily treated at Can Tho Pediatric Hospital, with 66.7% experiencing secondary infections. Warning signs included high fever, rapid pulse, abdominal pain (54.4%), nausea/vomiting (43.2%), and bleeding (5.7%). DEN‐1, DEN‐2, and DEN‐4 circulated during the study, with DEN‐1 predominating in 2018 (85.7%) and both DEN‐1 and DEN‐2 as primary causes in 2019 (52.2% and 39.1%, respectively). The DEN‐2 serotype and secondary infections may significantly contribute to severe dengue, and further research on dengue immunity is essential for understanding the effects of dengue vaccination.

## 1. Introduction

Dengue is a significant public health issue in many tropical and subtropical countries. Dengue fever (DF) has experienced a dramatic increase worldwide in recent decades. As of April 30, 2024, 90 countries have reported active dengue transmission. Over 7.6 million dengue cases have been reported to the WHO, including 3.4 million confirmed cases, 16,000 severe cases, and over 3000 deaths [1, 2]. DF was first reported in Vietnam in the late 1950s and has since become endemic in the country [3]. The number of reported cases varies significantly from year to year. Dengue outbreaks are more frequent in southern Vietnam, occurring year‐round with a seasonal peak during the rainy months from June to December. According to the national data, 85% of the nationwide infected cases and deaths are from the southern region [4, 5].

Can Tho is the fourth‐largest city in Vietnam, located in the Mekong Delta region. It has an approximate population of 1.5 million. The climate features two seasons (rainy and dry) and is affected by climate change. It has the highest reported incidence of dengue in Vietnam [6, 7].

The National Dengue Control Program, established in Vietnam in 1999, has successfully reduced mortality and morbidity nationwide. However, climate change, which is now associated with shifting seasonal patterns, may affect the clinical features of dengue infection. Recently, the Qdenga vaccine (TDV/TAK‐003, Germany) was first approved in Vietnam in May 2024 as a new and innovative method for preventing the public health threat of dengue [8, 9]. It began widespread immunization in September 2024, which may alter the pattern of the dengue epidemic in Vietnam later. Our study was conducted in the Can Tho City from 2018 to 2019 to determine the frequency and characteristics of DF in patients with acute febrile illness and to identify the factors associated with severe disease.

## 2. Materials and Methods

### 2.1. Study Population

This cross‐sectional study included 123 individuals admitted to Can Tho General Hospital and Can Tho Pediatric Hospital between 2018 and 2019 with an initial diagnosis of DF, following the Ministry of Health (MOH) guidelines for preventing and managing dengue hemorrhagic fever. These patients were hospitalized and provided blood full blood count testing and serum for the dengue antigen rapid test (NS1).

We included the blood count results in the collection forms and gathered serum from all study participants. The serum was kept at −80°C and then sent to the National Institute of Hygiene and Epidemiology (NIHE) in Hanoi. At NIHE, the samples were stored at −80°C until they were tested.

### 2.2. Ethics Statement

This study received approval from the NIHE Institutional Review Board (IRB‐VN01057‐11/2018, 15 May 2018). Informed consent forms were obtained from all adult subjects or a parent or guardian of children under 15 years of age. All data were anonymized.

### 2.3. Serology Tests

Serum samples were tested for dengue‐specific IgM and IgG using Dengue ELISA IgM Capture and Dengue ELISA IgG (Vircell Microbiologists, Spain), following the manufacturer’s instructions. ELISA test results are quantified using the antibody index. The antibody index is calculated using the formula: (sample OD/cutoff serum mean OD) × 10. An antibody index of > 11 indicates a positive result, an equivocal result falls within the range of 9–11 antibody index, and a sample with an antibody index below nine is considered negative.

### 2.4. Molecular Test

Serum samples were used to extract viral RNA with the Qiagen Viral RNA Mini Kit (Qiagen, USA). Real‐time reverse transcription‐quantitative PCR (qRT‐PCR) was then performed using published primer sets specific to the four dengue serotypes (DEN1, DEN2, DEN3, and DEN4) with a SuperScript™ III Platinum One‐Step Kit (Thermo Fisher Scientific, USA) according to the kit instructions. The primer and probe sequences that were published were used as follows [10] (see Table 1).

**TABLE 1:** Nucleotide sequence of primers and probe used in the qRT‐PCR assay [10].

Primer/probe		5′ fluor	Sequence (5′–3′)	3′ quencher	Gene
D1	D1F		CAA AAG GAA GTC GYG CAA TA		NS5
	D1C		CTG AGT GAA TTC TCT CTG CTR AAC		NS5
	Probe D1	FAM	CAT GTG GYT GGG AGC RCG C	BHQ1	NS5

D2	D2F		CAG GCT ATG GCA CYG TCA CGA T		E
	D2C		CCA TYT GCA GCA RCA CCA TCT C		E
	Probe D2	HEX	CTC YCC RAG AAC GGG CCT CGA CTT CAA	BHQ1	E

D3	D3F		GGA CTR GAC ACA CGC ACC CA		prM
	D3C		CAT GTC TCT ACC TTC TCG ACT TGY CT		prM
	Probe D3	Texas red	ACC TGG ATG TCG GCT GAA GGA GCT TG	BHQ2	prM

D4	D4F		TTG TCC TAA TGA TGC TRG TCG		prM
	D4C		TCC ACC YGA GAC TCC TTC CA		prM
	Probe D4	Cy5	TYC CTA CYC CTA CGC ATC GCA TTC CG	3IAbRQSp	prM

Briefly, qRT‐PCR was performed for a multiplex RT‐PCR reaction. About 5 μL of RNA was mixed with the following reagents: 2.2 μL of nuclease‐free H_2_O, 12.5 μL of 2× premix, 0.5 μL of forward and reverse primers for DENV‐1 and DENV‐3 (final concentration 1 μM), 0.25 μL of forward and reverse primers for DENV‐2 and DENV‐4 (final concentration 500 nM), 0.45 μL of each TaqMan probe (final concentration 180 nM), and 0.5 μL of SuperScript III RT/Platinum Taq to reach a final reaction volume of 25 μL.

The ABI 7500 FAST thermocycler (Applied Biosystems, USA) was used with standard cycling conditions: reverse transcription (RT) at 50°C for 30 min, RT inactivation at 95°C for 2 min, and fluorescence detection through 45 cycles of 95°C for 15 s and annealing at 60°C for 1 min. Amplification curves were evaluated by serotype, with the threshold line placed above the background signal, usually intersecting the initial exponential phase of each curve. Amplification curves with Ct values over 35 were considered erratic and negative.

### 2.5. Result Interpretation

The patients were classified as having DF, dengue with warning signs, and severe dengue, following the WHO Dengue Guidelines and the MOH guidelines for preventing and managing dengue hemorrhagic fever [11, 12].

Individuals were grouped into primary or secondary infection categories based on their dengue IgG, IgM, and qRT‐PCR results. If they tested positive for qRT‐PCR or IgM against DENV, or were single‐positive for qRT‐PCR or IgM only, they were classified as having a primary infection. Those with a high concentration of IgG antibodies (antibody index > 11) in serum and/or additional positive qRT‐PCR or IgM antibodies against DENV were classified as having a secondary infection.

## 3. Results

### 3.1. Study Population Characterization

Between May 2018 and October 2019, a total of 123 dengue infections were admitted to two hospitals in the Can Tho City. The number of patients was 62 in 2018 and 61 in 2019. Male patients accounted for 68 (55.3%) individuals, while female patients numbered 55 (44.7%). Of these, 111 patients (90.2%) were at the Can Tho Pediatric Hospital, and 12 patients were at the Can Tho City Hospital, corresponding to a primary age rate of approximately 81.3% for the 6‐ to 15‐year‐old group (Table 2). Most patients were admitted to the hospital with an initial diagnosis of DF (119 patients/96.7%), they were unaware of their DF infection history (70 patients/56.9%) (Table 2), all of the patients, 100% recovered, and the mortality rate was 0% (Table 2).

**TABLE 2:** Study population’s characterization.

Study population (*N* = 123)			Rate (%)
Gender	Male	68	55.3
	Female	55	44.7

Age	< 5	9	7.3
	6 to 15	100	81.3
	16 to 40	12	9.8
	> 60	2	1.6

Year	2018	62	50.4
	2019	61	49.6

Hospital	Can Tho City General	12	9.8
	Can Tho pediatric	111	90.2

Initial diagnosis	Dengue fever	119	96.7
	Dengue fever more than 7 days	1	0.8
	Others	3	2.5

Dengue fever history	Yes	38	30.9
	No	15	12.2
	Unknown	70	56.9

Hospitalization stay (day)	3	1	0.8
	4	11	8.8
	5	20	16.3
	6	18	14.6
	7	28	22.7
	8	25	20.3
	9	8	6.5
	10	5	4.1
	> 11	7	5.9

Treatment outcome	Discharge	123	100
	Death	0	0

#### 3.1.1. Clinical Presentations at the Time of Admission to the Hospital

All patients presented with fever at the time of hospital admission, most of whom had a temperature between 37°C and 39°C (106 patients), while 17 patients had a temperature exceeding 39°C. In addition to fever, a pulse rate of more than 100 beats per minute was detected in 24 individuals (19.5%) (Table 3). The warning signs of dengue infections were highlighted; our results showed that abdominal pain was reported in 67 individuals (54.4%), while nausea and/or vomiting were identified in 53 individuals (43.1%), and bleeding was noted in 7 patients (5.7%) (Table 3). Additional tests were conducted to assess the warning condition, which included platelet counts of fewer than 1000 cells/mm^3^ found in 110 individuals (89.4%), hematocrit levels increasing by more than 45% in 12 individuals (8.8%), and the tourniquet test to determine a patient’s hemorrhagic tendency, which was positive in 59 individuals (47.9%) (Table 3).

**TABLE 3:** Clinical manifestations of hospitalized patients with dengue infection.

Clinical manifestation		Number (*n*)	Rate (%)
Temperature (°C)	37 to 38	54	43.9
	> 38 to 39	52	42.3
	> 39	17	13.8

Pulse number/min	70–100	99	80.5
	> 100	24	19.5

Hematocrit	≥ 45%	12	8.8
	Less than 45%	111	90.2

Platelet (cell/mm^3^)	Less than 10000	110	89.4
	≥ 10000	13	10.6

Nausea/vomiting	Yes	53	43.1
	No	34	27.6
	Not clear	36	29.3

Abdominal pain	Yes	67	54.5
	No	56	45.5

Purpura (tourniquet testing)	Positive	59	47.9
	Negative	37	30.1
	Not clear	27	22.0

Bleeding	Yes	7	5.7
	No	72	58.5
	Not clear	44	35.8

### 3.2. Dengue Infection Category Identification

We use ELISA serology tests to categorize dengue infections based on the presence of IgM and IgG antibodies against dengue viruses. Our results showed a positive IgM antibody rate of 88.6% (109 individuals) and an IgG positivity rate of 66.7% (82 individuals). This indicates that the secondary infection rate is 66.7%, while the primary infection rate is 29.2%. Four sera tested negative for both IgM and IgG antibodies against dengue (Table 4).

**TABLE 4:** ELISA analysis of IgM and IgG results along with dengue classification.

ELISA results	Number (*n*)	Dengue category	Rate (%)
IgM (+), IgG (+)	73	Secondary infections	66.7
IgM (−), IgG (+)	9	Secondary infections	

IgM (+), IgG (−)	36	First infection	29.2

IgM (−), IgG (−)	5	Negative	4.1

### 3.3. Association of Dengue Serotypes and Categories With the Clinical Classification of Dengue Infection

The dengue infection can develop into a severe case, with warning signs influenced by several factors. Our study focused on dengue serotypes and categories; the results showed that serotypes DEN‐1, DEN‐2, and DEN‐4 circulated in the Can Tho city during the study period. DEN‐1 was the leading cause of the outbreak in 2018 (85.7%), while DEN‐1 and DEN‐2 were the main causes in 2019; however, DEN‐2 was predominant with a rate of 52.2%, and DEN‐1 followed with 39.1% (Table 5).

**TABLE 5:** Association of dengue classification and dengue serotypes.

**Index**		**2018 (*n* = 62)**		**2019 (*n* = 61)**	

Hospital stay (day)	Median	5.4		7.7	
	Longer	12		23	
	Shorter	4		4	

		**Number (*n*)**	**(%)**	**Number (*n*)**	**(%)**

Dengue serotype (*N* = 37)	DEN‐1	12	85.7	9	39.1
	DEN‐2	2	14.3	12	52.2
	DEN‐3	0	0	0	0
	DEN‐4	0	0	2	8.7
	**Total**	**14**	**100**	**23**	**100**

		**Number (*n*)**	**(%)**	**Number (*n*)**	**(%)**

Dengue classification [13]	Dengue	33	53.2	25	40.8
	Dengue warning sign	24	38.7	21	34.4
	Severe dengue	5	8.1	15	24.8

The analysis of hospital stays for all patients with dengue infection found that the median stay was 5.4 days in 2018, ranging from 4 to 12 days, while the median stay was 7.7 days in 2019, with a range from 4 to 23 days (Table 5).

In this study, following the WHO guidelines of 2009, the proportions of patients with DF, dengue with warning signs, and severe dengue were 33 (53.2%), 24 (38.7%), and 5 (8.1%) in 2018, and 25 (40.8%), 21 (34.4%), and 15 (24.8%) in 2019 (Table 5). Of these, the distribution of DF by primary infection and secondary infection was 11 (33.3%): 21 (66.7%) in 2018, and 7 (29.2%): 16 (70.8%) in 2019.

In the analysis, dengue warning signs were observed in 12 patients (50%) in 2018 and in four patients (19.1%) in 2019, indicating primary infection. In secondary infections, there were 12 patients (50%) in 2018 and 16 patients (89.9%) in 2019, respectively (Table 6).

**TABLE 6:** Association of dengue categories and dengue classification.

Dengue classification [13]	2018 (N = 61)				2019 (N = 57)				Negative	
	1st infection		2nd infection		1st infection		2nd infection			
	Number (*n*)	Rate (%)	Number (*n*)	Rate (%)	Number (*n*)	Rate (%)	Number (*n*)	Rate (%)	Number (*n*)	Rate (%)
Dengue	11	33.3	21	66.7	7	29.2	16	70.8	2	40
Dengue warning sign	12	50	12	50	4	19.1	16	80.9	1	20
Severe dengue	0	0	5	100	2	14.3	12	85.7	2	20

Severe dengue was not observed among patients in 2018; however, two patients (14.3%) were identified in 2019, who were indicated as primary infection. Among the secondary infection group, severe dengue was determined in five patients (100%) in 2018 and 12 patients (85.7%) in 2019 (Table 6).

Among the group of five negative patients (negative with all dengue tests), DF was indicated in two patients (40%), warning signs were observed in one patient (20%), and severe dengue was noted in two patients (40%) (Table 6).

## 4. Discussion

The Vietnam National Dengue Control Program was established in 1999 with the aim of reducing the number of dengue cases, decreasing the number of dengue‐related deaths, and preventing large‐scale dengue epidemics. However, the number of dengue cases remains high. It has caused a substantial health and economic burden in Vietnam [3, 14, 15]. Our study population consisted of 123 dengue patients enrolled over a 20‐month period, from May 2018 to October 2019. Of these, 111 patients (90.2%) had been treated at the Pediatric Can Tho Hospital; the results indicated that children under 15 years old may be more susceptible than the adult population in Can Tho city. This finding provides further evidence for the study of the age distribution of dengue clinical cases in South Vietnam from 2000 to 2015, with the mean age of dengue cases ranging from 12.2 (8.8 years) to 16.8 (13.3 years) [16].

### 4.1. Clinical Manifestations of Warning Signs

Our study focused on the clinical features of dengue warning signs and severe dengue at the time of hospital admission, which helps doctors predict the outcome and provide an approximate treatment. Based on the guidelines of the WHO, the dengue warning signs considered included abdominal pain, persistent vomiting, clinical fluid accumulation, mucosal bleeding, lethargy, liver enlargement, and laboratory results showing an increase in hematocrit concurrent with a rapid decrease in platelet count [13]. However, our study collected manifestations upon admission; thus, our findings indicate that nausea or vomiting was noted at a rate of 43.1%, abdominal pain at 54.4%, and bleeding was reported at only 5.7% (Table 3). Another findings showing a decrease in platelets less than 10.000 cell/mm^3^ and an increase in hematocrit more than 45% were observed at 89.4% and 8.8%, respectively (Table 3). These findings were correlated to a study in Indonesia indicating that abdominal pain and persistent vomiting are common warning signs of dengue infection in children aged 1–14 years [17]. Following the WHO guidelines published in 2009, our study classified a total of 45 dengue patients (36.5%) with warning signs and 20 patients (16.2%) in severe condition at the time of admission to the hospital, most studies use clinical classification for dengue infections during hospital stays; however, our study conducts early evaluations to reduce mortality and enhance treatment effectiveness. Additionally, the WHO uses ALT and ALS values greater than 1000 U/L as criteria for severe dengue; our results showed that only one case had an AST value exceeding 1000 U/L (data not shown), which was associated with dengue shock syndrome [13]. Although this factor is uncommon in our study, early assessment of live enzyme levels may serve as a predictive marker for dengue severity.

#### 4.1.1. The Classification of Dengue Was Affected by Dengue Serotypes and Infection History

We analyzed the pathogens (dengue serotypes) and the infection history of dengue patients, which correlated with disease severity and treatment outcomes. The significant finding is the higher rate of DEN‐2 in 2019 (52.2%) compared to 2018 (14.3%), along with an increase in severe dengue cases, which rose from 5 cases (8.1%) in 2018 to 15 cases (24.8%) in 2019. This may have led to a longer median hospital stay of 7.7 days in 2019, compared to 5.4 days in 2018 (Table 4). Our results resemble those of a recent study conducted at the Can Tho Children’s Hospital from October 2022 to March 2023, which reported a higher rate of dengue warning signs (42.7%) and severe dengue (29.2%) caused by DEN‐2, while DEN‐1 caused 15.6% of dengue warning signs and 8.3% of dengue severe [7]. Another report in Singapore between April 2005 and December 2011 showed that the risk of developing DHF and SD in DEN‐1 patients was higher compared to DEN‐2 or DEN‐3 patients [18]; however, this study focused on adult patients with a median age of 35–37 years; thus, the presence of clinical features may account for the different findings of our study. Nevertheless, further genetic characterization of DEN serotypes is needed to understand the pathogenicity of circulating viruses.

Another factor identified as a key aspect of severe dengue is the history of dengue infection [4, 19, 20]. We conducted serology testing to determine IgM and IgG antibodies from serum collected on the day of admission, and the results indicated categories of dengue infection. Our findings showed that 66.7% of patients had a secondary infection, 29.2% had a primary infection, and 4.1% were serology negative (Table 4). The incidence of dengue secondary infection that matched the dengue warning signs was 50% in 2018, while in 2019, it rose to 80.9%. Additionally, 100% of severe cases were identified as secondary infections in 2018, compared to 85.7% in 2019 (Table 6).

Since the Qdenga vaccine (Takeda, Japan) was licensed in Vietnam in May 2024 and rolled out nationwide in September of the same year, it is recommended for individuals aged 4 years and older who have experienced a past dengue infection [8, 21]. Our findings suggest that serology testing should be conducted before dengue vaccination for children under 15 years old to confirm previous dengue infection, as the rate of primary infection is 29.2% (Table 4).

Our results indicate that the risk of severe dengue in children is associated with the DEN‐2 serotype and secondary dengue infection. This finding provides an immune profile of dengue infection in the Can Tho City, Vietnam, which may help assess the effectiveness of the Qdenga vaccine and monitor dengue circulation in Vietnam in the future.

The limitation of the study is that it does not analyze features during the treatment period; however, the outcomes of the treatment were recorded for all discharged patients. Another limitation is the lack of genetic analysis of dengue viruses causing diseases, and further characterization should be done to provide a clear answer regarding the association between dengue serotypes and severe disease.

## 5. Conclusion

Patients hospitalized for dengue in the Can Tho City between 2018 and 2019 were primarily children under 15 years. The DEN‐1 and DEN‐2 serotypes are the leading causes of infection, with the DEN‐2 serotype and secondary infections potentially playing a critical role in severe dengue. Further research on dengue immunity and active surveillance of dengue infections is necessary to evaluate the efficacy and other associated factors of the Qdenga vaccine in relation to the dengue epidemic in Vietnam.

## Funding

This research was supported by the Japan Agency for Medical Research and Development (AMED) under Grant number JP15fm010800 and the Ministry of Science and Technology, Vietnam (MOST) under Grant number NDT/e‐ASIA/23/06.

## Conflicts of Interest

The authors declare no conflicts of interest.

## Data Availability

The data that support the findings of this study are available on request from the corresponding author. The data are not publicly available due to privacy or ethical restrictions.
